# The presence and degradation of nerve fibers in articular cartilage of neonatal rats

**DOI:** 10.1186/s13018-022-03221-2

**Published:** 2022-06-27

**Authors:** Zheng Wang, Bin Liu, Kaifeng Lin, Chunguang Duan, Chunmei Wang

**Affiliations:** 1 Department of Orthopedics, General Hospital of Northern Theater Command, Shenyang, 110016 Liaoning China; 2 Department of Orthopedics, The 900 Hospital of Joint Logistic Support Force, Fuzhou, 350025 Fujian Province China; 3 Department Spine, Shenzhen University General Hospital, Shenzhen, 518055 Guangdong China; 4 Department of Orthopaedics, Xijing Hospital, Air Force Medical University, Xi’an, 710032 Shanxi China

**Keywords:** Articular cartilage, Nerve fiber, Neuropeptide, Chondrocyte

## Abstract

**Purpose:**

To investigate the presence and change of nerve fibers and neuropeptide during early development of articular cartilage in neonatal rats.

**Methods:**

Articular cartilage in distal-femoral epiphyses was collected from neonatal Sprague Dawley rats, which were 1-day, 5-day, and 10-day postnatal (P1, P5 and P10). Microscopy, immunofluorescence, transmission and scanning electron microscopy (TEM and SEM) were performed for detection of nerve fibers. Quantitative analysis for substance P (SP) and neuropeptide Y (NPY) was conducted using immunofluorescence and enzyme-linked immunosorbent assay (ELISA).

**Results:**

TEM showed the existence of myelinated nerve fibers in the extracellular matrix of articular cartilage in both P1, P5 and P10 rats, and they formed synaptic contacts with chondrocytes. During this time, chondrocytes proceeded with their development, and the nerve fibers gradually degraded. The ELISA results showed significant increase of the sensory neuropeptide SP and the sympathetic neuropeptide NPY in the cartilage tissue. Immunofluorescence results showed the distribution of SP and NPY in the perichondrium, the cartilage canals, the plasma of chondrocytes, and extracellular matrix in the cartilage tissue.

**Conclusions:**

Nerve fibers exist in the matrix of articular cartilage during early development of knee joints in neonatal rats. Nerve fibers form synaptic contacts with chondrocytes at the early stage and then degrade gradually in the course of chondrocyte development. SP and NPY significantly increase in articular cartilage during this very period. These results indicate that the nerve fibers and the neuropeptide they secrete may exert important effect on the development of articular cartilage.

## Introduction

Articular cartilage (AC) is traditionally considered as avascular and devoid of nerves. However, there is sparse evidence showing the existence of nerve fibers in certain area of AC at specific time period. Hedberg et al. [1] demonstrated the transient local presence of nerve fibers in cartilage canals at onset of secondary ossification in the rat knee joint. Schwab and Funk [2] described that, in the adult rat knee, nerve fibers are frequently present in the periosteum and some fine nerve fiber branches penetrate into the outer layers of AC. Furthermore, in human, nerve fibers were also found in superficial zone of adult sacroiliac joint cartilage and cartilage canals in fetal and infant joints [3, 4]. Collectively, nerve fibers have been found in perichondrium, cartilage canals, outer layers of articular and metaphyseal cartilage and osteoarthritic AC [4–8], and all the discovered nerve fibers were inevitably unmyelinated. Except those short penetrations from perichondrium or cartilage canals, no nerve fiber has been found in the matrix of AC. However, it was also suggested that the sample preparation procedure and limited detecting technique might cause omission for potential discovery [2, 8].

There have been few reports concerning the relationship of cartilage development and nerve fibers or the effect of nerve fibers on cartilage development. Schwab et al. found that nerve fibers from periosteum near AC do not only enter the hyaline cartilage, but also come in close contact to adjacent chondrocytes with a distance of less than 1 μm [6]. Previous reports described the calcitonin gene-related peptide (CGRP)-, substance P (SP)-, neurokinin A (NKA)- or neuropeptide Y (NPY)-immunoreactivity of the nerve fibers contacting the chondrocytes [2, 6, 8]. These neuropeptides have been reported to have multiple effects on the development and pathophysiology of chondrocytes and AC [5, 9]. These findings indicated the potential effect of the nerve fibers on the development and pathophysiology of AC. Nevertheless, it is still unclear how nerve fibers interact with chondrocytes and AC and what effect they exert on the development and pathophysiology of chondrocytes and AC. Present study aims to investigate the presence of nerve fibers in AC and their relationships, in order to contribute to the research of cartilage development and AC repair and regeneration.

## Materials and methods

### Animal preparation

Twenty-four neonatal Sprague Dawley rats (from Experimental Animal Center of Fourth Military Medical University, Xi’an, China) were obtained for sample collection at 1-day postnatal (P1, *N* = 8), 5-day postnatal (P5, *N* = 8) and 10-day postnatal (P10, *N* = 8). Briefly, pentobarbital (0.1% solution, 30 mg/kg) was used for anesthesia, followed by perfusion with 4% paraformaldehyde injection via left ventricle according to reported protocol [10]. The knee joints of hind limbs (*N* = 16 in each group) were dissected and collected. All the chemicals were purchased from Sigma-Aldrich (St. Louis, United States) unless noted specifically. This study conformed to the requirements of the ethics committee of General Hospital of Northern Theater Command(Y-2022-010).

### Hematoxylin–eosin staining (HE) and microscopic observation

The knee joints (*N* = 3 in each group) were fixed in 4% paraformaldehyde solution for 3 days and decalcified in ethylenediaminetetraacetic acid (EDTA, 10%) solution for four weeks, then dehydrated in graded concentrations of ethanol, and embedded in paraffin, and cut into serial 6-μm-thick sections. The sections were deparaffinized and rinsed by distilled water and then stained with hematoxylin–eosin. Finally, the sections were observed under optical microscope (Leica DM6000B, Leica Microsystems GmbH Co. Ltd., Wetzlar, Germany) for the location of the cartilage tissue and conditions of the chondrocytes.

### Scanning electron microscopy (SEM)

Cartilage tissue samples (*N* = 3 in each group) were cut out in 1 mm^3^ from the center of each perfused knee joint. The samples were fixed with glutaraldehyde solution (2.5%), dehydrated in graded concentrations of ethanol and desiccated. At last, the samples were coated with gold and observed under SEM (HITACHI S-3400, Chiyoda, Tokyo, Japan) for the morphology of the cartilage tissue and conditions of the chondrocytes.

### Transmission electron microscopy (TEM)

Cartilage tissue samples (*N* = 3 in each group) were cut out in 1 mm^3^ from the center of each perfused knee joint. After fixation in glutaraldehyde solution (2.5%), the samples were treated with osmium tetroxide solution (1%) for two hours followed by propylene oxide for 10 min and subsequently embedded in epoxy resin. Each embedded sample was cut into 50–70 nm slices by ultramicrotome and stained with uranyl acetate and lead citrate. Finally, the ultrathin sections were observed under TEM (FEI TECNAI G2, Hillsboro, United States) to examine nerve fibers’ existence in the cartilage tissue and the development of both the chondrocytes and the AC matrix.

### Immunofluorescence (IF)

The knee joints (*N* = 3 in each group) were fixed in 4% paraformaldehyde solution for 3 days and decalcified in EDTA (10%) solution for four weeks, then dehydrated in graded concentrations of ethanol, embedded in paraffin and cut into serial 6-μm-thick sections. The paraffin sections were deparaffinized, blocked by goat serum and Triton-X 100 transparent film, and incubated with rabbit-anti-rat anti-NPY (Abcam, Cambridge, USA) or rabbit-anti-rat anti-SP (Abcam, Cambridge, USA) primary antibodies at 37 °C for 30 min. Then, the samples were rinsed with 0.01 M PBST for three cycles of 5 min and incubated with goat-anti-rabbit FITC fluorescence secondary antibody or goat-anti-rabbit rhodamine fluorescence secondary antibody in 37 °C dark for 30 min. After incubation, the samples were rinsed with 0.01 M PBST in dark for three cycles of 5 min and mounted with glycerol buffer. At last, the samples were observed under confocal laser scanning microscope (Olympus Fluo View FV-1000, Olympus Corporation, Tokyo, Japan). Semi-quantitative analyses for the IF images were performed with analysis software FV10-ASW 3.1 (Olympus Corporation, Shinjuku, Tokyo, Japan).

### Enzyme-linked immunosorbent assay (ELISA) for SP and NPY

Knee joints in different rat groups (*N* = 4 in each group) were harvested. The cartilage tissues were cut into same volume and size and then added into phosphate-buffered saline in a homogenizer for complete homogenization. The mixture was centrifuged for 15 min (2000–3000 r/min), and then, the supernatant was collected. The expression of SP and NPY in the supernatant of cartilage tissue was examined with ELISA Kit (RayBiotech Life, Norcross USA). The absorbance was detected at 450 nm with a microplate spectrophotometer (Tecan Infinite M2000, Tecan Group Ltd., Männedorf, Zürich, Switzerland).

### Statistical analysis

Student’s t test was performed for comparisons between two groups. For comparison, more than two groups, one-way ANOVA and post hoc analysis, were used. Differences with *P* < 0.05 were considered statistically significant.

## Results

### Microscopic observation

**Fig. 1:**
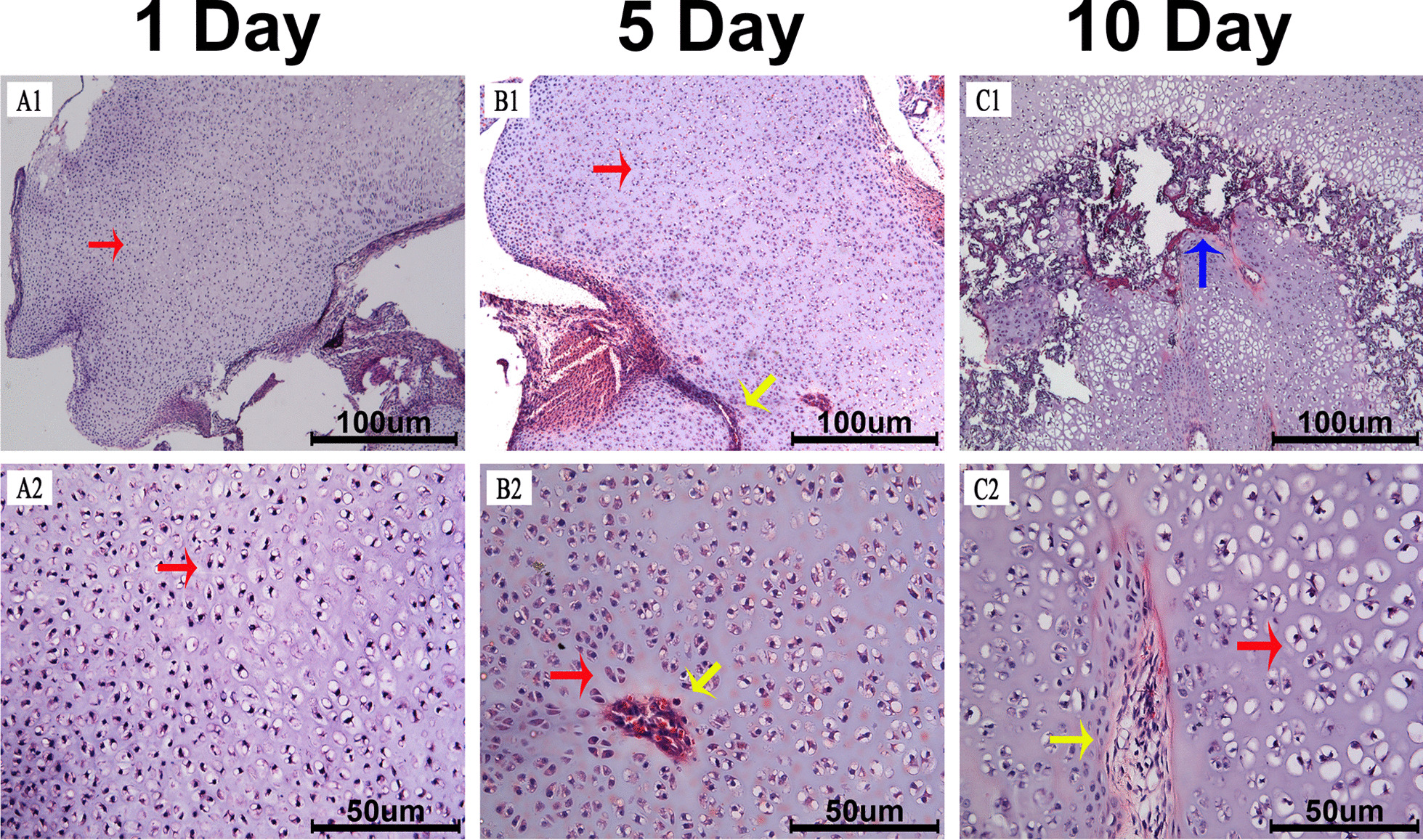
Microscopy of the cartilage tissue. **A1**, **B1**, **C1** articular cartilage (AC) of 1-, 5- and 10-day postnatal rats. Red arrows point the chondrocytes, yellow arrow represents the cartilage canal, and the blue arrow represents the secondary ossification center. **A2** chondrocytes with few calcium depositions in the AC of 1-day postnatal rats. **B2** a cartilage canal containing blood vessels in the AC of 5-day postnatal rats (yellow arrow) with increased calcium deposition. **C2** cartilage canals connected secondary ossification center in the AC of 10-day postnatal rats (yellow arrow)

In the AC of neonatal rats, chondrocytes densely resided in the parenchyma of the cartilage tissue in each group (Fig. 1A1, B1, C1). Cartilage canals containing blood vessels existed in the center of P5 and P10 rats’ AC (Fig. 1B1–2). In P10 rats, cartilage canals connected secondary ossification centers (Fig. 1, C1–2). During this period, the chondrocytes developed with gradual differentiation and increased calcium deposition in the matrix (Fig. 1, A2, B2, C2).

**Fig. 2:**
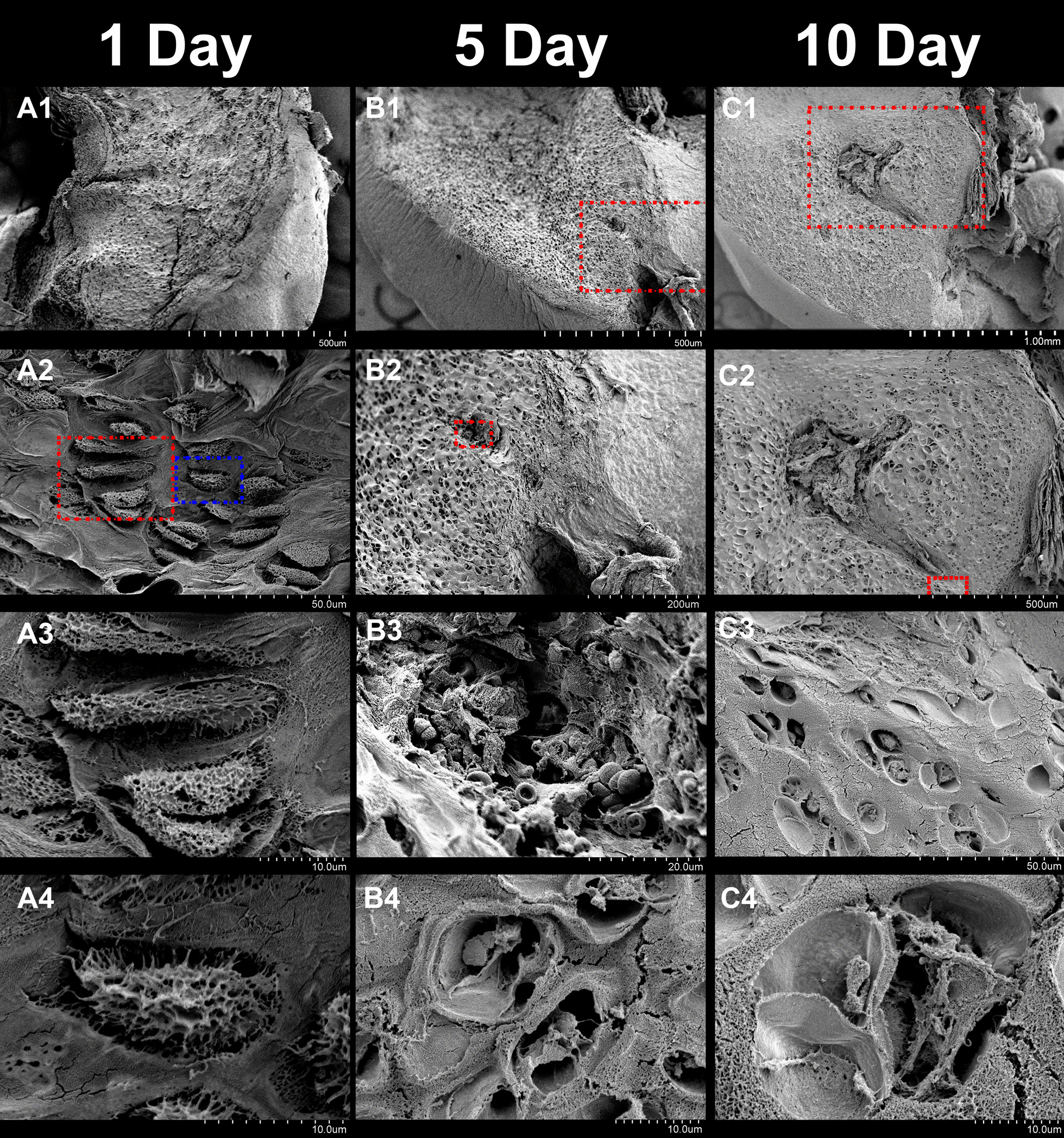
Scanning electron microscopy of the articular cartilage in 1-day (**A1**–**4**), 5-day (**B1**–**4**) and 10-day (**C1**–**4**) postnatal rats. **A1**, **B1**, **C1** parenchyma of the articular cartilage (AC) in each group. **A2** the chondrocytes densely reside in the AC. **A3**, extracted from the red box in **A2** typical face-to-face structure of the chondrocytes. (**A4**, extracted from the blue box in **A2**) The chondrocytes are in fine activity with complete cellular structure and abundant cell processes. (**B2**, extracted from the red box in **B1**) the cartilage canal (red box). (**B3**, extracted from the red box in **B2**) vascular wall and erythrocytes in the cartilage canal. (**B4**) The chondrocytes are in poor activity with few cell processes. (**C2**, extracted from the red box in **C1**) The secondary ossification center is connected with cartilage canals. (**C3**, extracted from the red box in **C2**) The cartilage canal is surrounded by abundant chondrocytes. **C4** the chondrocytes and rough surface of the matrix with much calcium deposition

### SEM observation

In P1 rats, the chondrocytes densely resided in the AC (Fig. 2, A1–2), and typical face-to-face position of the chondrocytes was also observed (Fig. 2, A3). The chondrocytes were in fine vitality with complete cellular structure and abundant cell processes on their surfaces. The extracellular matrix (ECM) had a smooth surface with abundant collagen fibers and little calcium deposition (Fig. 2, A4).

**Fig. 3:**
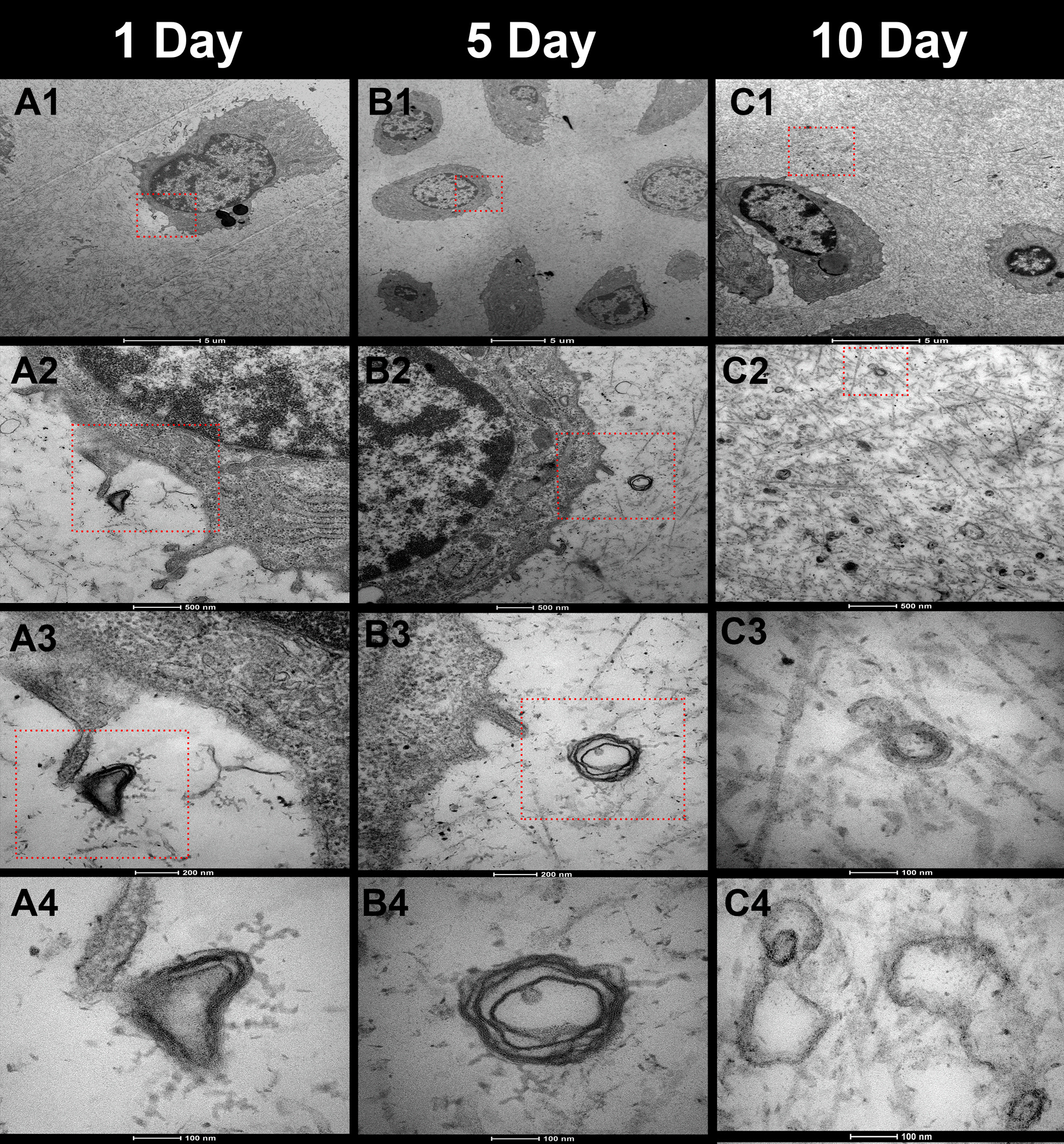
Transmission electron microscopy of the articular cartilage in 1-day (**A1**–**4**), 5-day (**B1**–**4**) and 10-day (**C1**–**4**) postnatal rats. **A1**, **B1**, **C1** chondrocytes and surrounding matrix in the articular cartilage (AC). (**A2**–**4**, extracted from the red boxes in each upper image) the nerve fiber around the chondrocyte, with typical lamellar structure of myelin sheaths consisting of 6–7 layers, and the contact between it and the chondrocyte. (**B2**–**4**, extracted from the red boxes in each upper image) a nerve fiber near a chondrocyte. The distance between nerve fibers and the cell processes of chondrocytes increased, and the medullary sheath has less layers (4–5) and looser structure. **C2** nerve fibers away from the chondrocytes. (**C3** extracted from the red boxes in **C2**, and **C4** from other area) even less layers (2–3) and looser structure of the myelin sheaths and signs of degradation of the nerve fibers

**Fig. 4:**
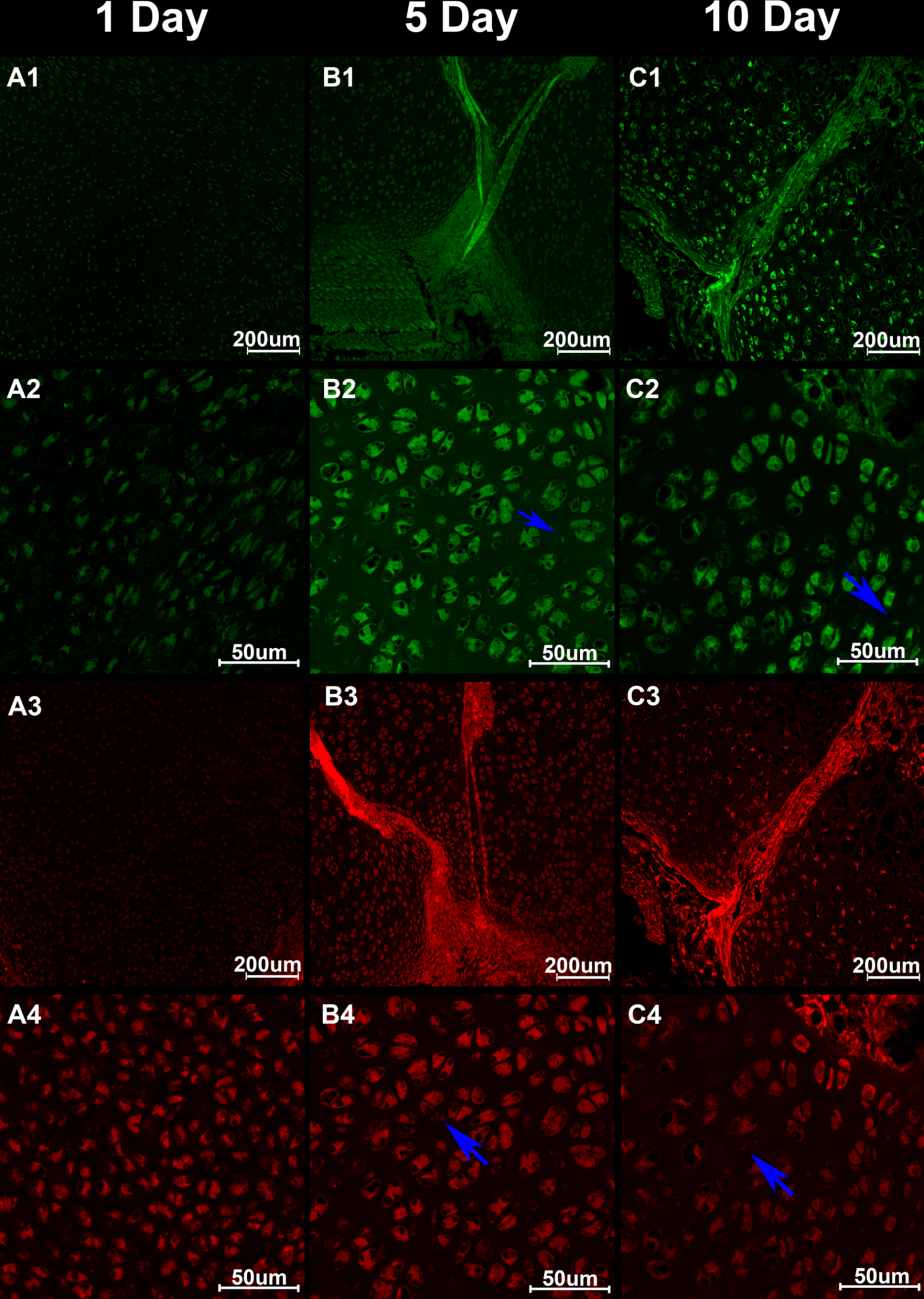
Immunofluorescence of the articular cartilage. The green fluorescence represents neuropeptide Y (NPY), and the red fluorescence represents substance P (SP). **A1**–**4** SP and NPY densely existed in the plasma of the chondrocytes in 1-day postnatal rats. Around the chondrocytes, no NPY and SP were found. **B1**–**4**, **C1**–**4** SP and NPY densely existed in the perichondrium, cartilage canals and the plasma of the chondrocytes in 5-day and 10-day postnatal rats. There are also NPY and SP expression in the matrix around the chondrocytes in the 5-day and 10-day rats (blue arrows)

In P5 rats, the cartilage canal could be observed (Fig. 2, B1–2). There were vascular walls and erythrocytes in the cartilage canal (Fig. 2, B3). With further magnification, it was observed that the chondrocytes were in decreased vitality with less cell processes (Fig. 2, B4). The ECM had rough surface with some calcium deposition.

In P10 rats, the secondary ossification centers, surrounded by chondrocytes, could be observed in the center of the cartilage tissue, exhibiting the endochondral ossification (Fig. 2, C1–2). The cartilage canal connected the secondary ossification center. The chondrocytes had incomplete cell structures and decreased cell processes (Fig. 2, C3–4). The ECM had rough surfaces with much calcium deposition.

### TEM observation

In P1 rats, there were certain collagen fibrils surrounding the chondrocytes and little calcium deposition in the AC (Fig. 3, A1). A fibrous structure was occasionally seen around the chondrocytes, which were suspected to be nerve fibers (Fig. 3A2–3). Observing this fibrous structure under a magnification of 135,000 (Fig. 3, A4), it could be confirmed as myelinated nerve fibers with typical lamellar structure of myelin sheaths consisting of 6–7 layers of Schwann cells. The nerve fibers formed close dispersive contact, which had a distance less than 100 nm and seemed to have secretory function, with the cell processes of chondrocytes.

In P5 rats, there were more collagen fibrils and calcium deposition in the AC matrix (Fig. 3B1). Nerve fibers still existed around the chondrocytes, but the distance between them increased (Fig. 3B2–3). Under magnification of 135,000, the typical lamellar structure of myelin sheaths could be observed with less layers (4–5) and looser structure (Fig. 3B4).

In P10 rats, there were furthermore collagen fibrils and calcium deposition in the AC matrix (Fig. 3C1). There were nerve fibers around the chondrocytes as well, with even farther distance (Fig. 3C2). Under magnification of 135,000, the typical lamellar structure of the myelin sheaths could be observed with even less layers (2–3), looser structure and signs of degradation (Fig. 3, C3–4).

### IF observation

**Fig. 5:**
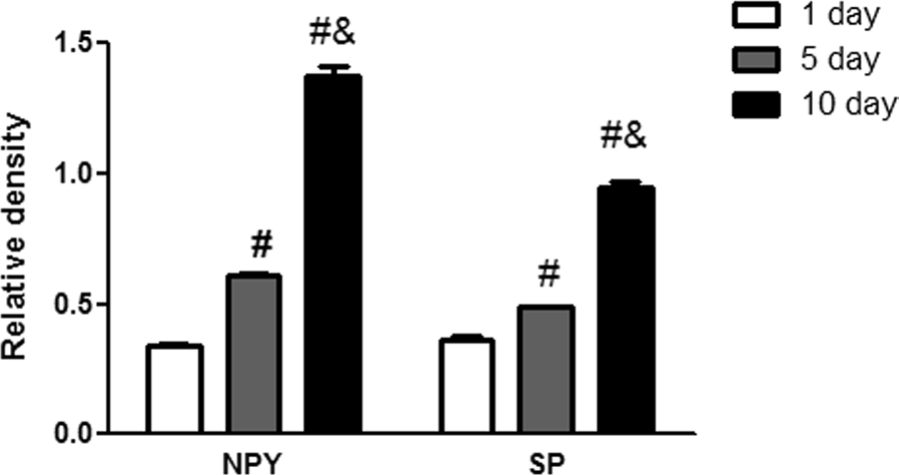
Semi-quantitative analysis of the expression of substance P (SP) and neuropeptide Y (NPY). #*P* < 0.05 compared with P1 group; &*P* < 0.05 compared with P5 group

According to the results of IF, SP and NPY densely existed in the perichondrium, cartilage canals and the plasma of the chondrocytes (Fig. 4, A1–C4). In P5 and P10 AC, there were SP and NPY in the matrix around the chondrocytes as well. Semi-quantitative analysis suggested that the expression of SP and NPY increased, while AC developed with time (Fig. 5).

### ELISA result of SP and NPY

**Fig. 6:**
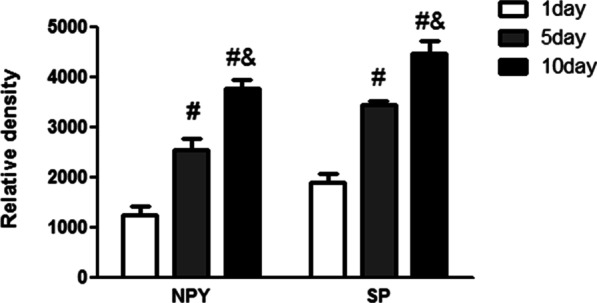
ELISA analysis of the expression of substance P (SP) and neuropeptide Y (NPY). #*P* < 0.05 compared with P1 group; &*P* < 0.05 compared with P5 group

The ELISA result suggested that the content of SP and NPY increased significantly in the AC of neonatal rat knee joints, while AC developed with time (Fig. 6).

## Discussion

Healthy adult cartilage is known to be devoid of blood vessels and nerve fibers, but the existence of nerve fibers in the developing cartilage has not been determined. It is hard to identify their existence under ordinary optic microscope because of the imperceptibility of the nerve fibers. Theoretically, the electron microscope can be used to observe, recognize and analyze the nerve fibers in an ultra-micro aspect. Current study made the ultrathin section of the articular cartilage during early development of the knee joints in neonatal rats and observed the ultrastructure of the nerve fibers. We found that the nerve fibers appeared around the chondrocytes in cartilage parenchyma and formed synaptic contacts with the chondrocytes in 1-, 5-, and 10-day postnatal SD rats. During this very period, the chondrocytes proceeded their development with enhanced secretion function, increased collagenous fibril, and extracellular calcium deposition, and gradually degraded nerve fibers. This is the first time that nerve fibers are found in the parenchyma of AC.

Previous reports described the existence of nerve fibers around or in the superficial layer of the AC [5]. The existence of nerve fibers has been identified in perichondrium, cartilage canals, epiphyseal plate, and the boundary between the perichondrium and AC, however, never in the AC matrix. Using TEM, we observed the existence of nerve fibers and their ultrastructure in the AC matrix around chondrocytes during AC development. The nerve fibers were myelinated and formed dispersive and seemingly secretory contacts with the cell processes of chondrocytes in the 1-day postnatal rats. The myelin sheath of nerve fibers lost its layers gradually with looser structure and decreased contacts with chondrocytes in the 5-day postnatal rats. Ten days after birth, the myelin sheath degraded significantly and the nerve fibers were even farther from the chondrocytes. These findings suggested that, following the AC development, the nerve fibers will degrade and may have a regulating effect on the development and differentiation of the chondrocytes because of their close contacts and potential interaction. Our findings also support the theory that the existence of nerve fibers during cartilage development is temporary [1].

By investigating the knee joints of 1-day postnatal rats using immunofluorescence, previous studies found the existence of CGRP and SP in the perichondrium, the cartilage canals, the epiphyseal plate, and the boundary between perichondrium and AC. Our immunofluorescence results showed that there were SP and NPY in the matrix around chondrocytes as well in the knee joints of either 5-day postnatal or the 10-day postnatal rats. Meanwhile, the fluorescence intensities of SP and NPY increased following the AC development. We hypothesize that nerve fibers in the matrix degrade gradually during AC development and release abundant peptides, increasing the immunofluorescence intensity so that we can observe the distributions of SP and NPY in the matrix around chondrocytes during AC development.

During the development of neonatal rats’ knee joint cartilage, the ELISA result showed increased contents of the sensory SP and sympathetic NPY, which is in consistence with the immunofluorescence results. The significantly increased expressions of SP and NPY suggest potential effect of neuropeptide on the chondrocyte development during the early development of knee joints in neonatal rats. In the studies on the effect of SP on chondrocytes in vitro, researchers have proved the strong expressions of SP and its receptor NK-1 in chondrocytes. In addition, Opolka A et al. [11] demonstrated that costal chondrocytes from newborn mice when stimulated with SP induced proliferation and cell–matrix adherence by stimulating formation of focal adhesion contacts. This observation implies that SP might modulate proliferation rate of growth plate chondrocytes and consequently terminal differentiation during endochondral ossification. It was also found that SP can increase the proliferation rate of chondrocytes via receptor NK-1 [7]. Other researchers also suggest that SP has autocrine functions and can modulate chondrocyte metabolism and cartilage homeostasis differentially during skeletal growth and in pathophysiology [12, 13]. There are few studies on the relationship between NPY and chondrocytes. Long H [14] demonstrated the existence of NPY ( +) nerve fibers during endochondral ossification in fracture healing and the promoting effect of NPY on proliferation of chondrocytes. The results of our present study, in combination with the above studies, suggest that NPY and SP can affect chondrocyte and AC development. Nevertheless, the specific effect and underlying mechanism demand further research.

Studies have proved that there are no nerve fibers in AC in normal adult humans. Nevertheless, nerve fibers are involved in the pathophysiology of the cartilage tissue. Suri and colleagues [8] have localized both sensory (SP- and CGRP-positive) and sympathetic (NPY-positive) nerve fibers in the articular cartilage in human tibiofemoral osteoarthritis. This study suggests that blood vessels and nerve fibers penetrate into the osteoarthritis lesion, which may affect the condition of chondrocytes. Beside the pain brought by this invasion, these invaded nerve fibers may exert other effects on the chondrocytes. Considering the findings from current study, nerve fibers in the cartilage parenchyma and its influence on AC development may provide AC repair with a new research direction.

## Conclusion

Myelinated nerve fibers exist in the parenchyma of articular cartilage during early development of knee joints in neonatal rats, and they form synaptic contacts with chondrocytes. Following the articular cartilage development, the nerve fibers degrade gradually. Meanwhile, substance P and neuropeptide Y increase in the cartilage during articular cartilage development, which may be released from nerve fiber degradation. These results indicate that the nerve fibers and the neuropeptide they secrete may exert important effect on the development of articular cartilage.

## Data Availability

The datasets are available from the corresponding authors on reasonable request.

## Abbreviations

SP: Substance P
NPY: Neuropeptide Y
ELISA: Enzyme-linked immunosorbent assay
AC: Articular cartilage
CGRP: Calcitonin gene-related peptide
SEM: Scanning electron microscopy
TEM: Transmission electron microscopy
IF: Immunofluorescence
